# A Comparison of the Minimum Inhibitory Concentration of Antibiotics in Staphylococcus Species Isolated From Orthopedic and Respiratory Medicine Infections

**DOI:** 10.7759/cureus.49535

**Published:** 2023-11-27

**Authors:** Akito Tomoyama, Naomi Kobayashi, Hyonmin Choe, Hiroyuki Ike, Yohei Yukizawa, Shota Higashihira, Shu Takagawa, Ken Kumagai, Yutaka Inaba

**Affiliations:** 1 Department of Laboratory Medicine, Yokohama City University Hospital, Yokohama, JPN; 2 Department of Orthopaedic Surgery, Yokohama City University Medical Center, Yokohama, JPN; 3 Department of Orthopaedic Surgery, Yokohama City University School of Medicine, Yokohama, JPN

**Keywords:** drug resistance, coagulase-negative staphylococci, implant, orthopaedic, minimum inhibitory concentration, staphylococcus species

## Abstract

Introduction: Antibiotic susceptibility is very important for the successful treatment of orthopedic infections, particularly for implant-related infections. While the minimum inhibitory concentrations (MICs) of *Staphylococcus* species were well investigated for the isolates from the respiratory tract, investigations for orthopedic pathogens are very limited. We investigated the antibiotic MIC values of *Staphylococcus *species isolated from orthopedic infections and compared them with those of respiratory medicine isolates used as a control.

Methods: The MICs of vancomycin (VCM), arbekacin (ABK), teicoplanin (TEIC), linezolid (LZD), and rifampicin (RFP) of a total of consecutive 259 (89 orthopedic and 170 respiratory) *Staphylococcus *speciesisolated in our laboratory from January 2013 to July 2016 were retrospectively reviewed. Differences between the MICs of each antibiotic in orthopedic and respiratory samples were determined.

Results: The number of methicillin-sensitive *Staphylococcus aureus* (MSSA) with a VCM MIC of <0.5 μg/mL among respiratory isolates was significantly higher than that among orthopedic isolates, while those with a MIC of 2 μg/mL were significantly lower (P = 0.0078). The proportion of methicillin-resistant coagulase-negative staphylococci (MRCNS) isolates with a VCM MIC of 2 μg/mL was significantly higher in orthopedic samples than that of methicillin-resistant *Staphylococcus aureus* (MRSA) (P < 0.001) in respiratory isolates. The proportion of MRCNS orthopedic isolates with an RFP MIC of >2 μg/mL was significantly higher (P = 0.0058) than that of other orthopedic staphylococci.

Conclusions: The VCM MICs of *Staphylococcus *species from orthopedic infections were higher than those of respiratory samples, particularly MRCNS from implant-related samples.

## Introduction

Infection is one of the most serious complications of orthopedic surgery, particularly in implant-related surgical procedures, such as total joint arthroplasty. Surgical debridement and antibiotic treatment is the primary approach in most cases [1], but a key challenge in the treatment of periprosthetic joint infection (PJI) is the failure of antibiotic treatment in methicillin-resistant staphylococci (MRS) [2]. Determining the minimum inhibitory concentration (MIC) of an antibiotic agent is the gold standard assessment for any infectious disease. The breakpoint of vancomycin (VCM) for *Staphylococcus aureus*, as defined by the Clinical and Laboratory Standards Institute (CLSI), is as follows: sensitive, ≤2 mg/L; intermediate, 4-8 mg/L; and resistant, ≥16 mg/L. In Japan, many medical institutions have adopted these CLSI breakpoints. However, when selecting an antibiotic in the clinical setting, reference is usually made only to sensitivity or resistance. Despite a positive result for drug sensitivity, there may be slight differences in actual susceptibility, or MIC, among species.

Increased incidence of methicillin-resistant *Staphylococcus aureus* (MRSA) in knee and hip PJI is recently reported, indeed [3]. Particularly in the case of MRS infection, not only drug sensitivity but also the MIC itself may be an important factor for treatment success [4]. Few studies have investigated differences in MIC in orthopedic surgery, including PJI, and we hypothesized that the MIC of *Staphylococcus* species in orthopedic isolates may be higher than the MIC of those from internal medicine because we frequently have difficulty in treating PJI only by antibiotics. Therefore, this study aimed to determine the MIC profile of a range of antibiotics against *Staphylococcus* species from orthopedic infection cases in comparison with respiratory medicine isolates as ordinary isolated control samples.

## Materials and methods

The retrospective, cross-sectional study design was approved by the local institutional review board. *Staphylococcus* species isolated in our laboratory from January 2013 to July 2016 were reviewed. All specimens from respiratory medicine were sputum. Orthopedic specimens included joint fluid, tissue, closed pus, puncture fluid, and other sterile specimens. Respiratory medicine is the most common department where staphylococci are detected; therefore, *Staphylococcus aureus* isolated in respiratory medicine departments were used as a reference control. Additional clinical information obtained from each orthopedic case included the original diagnosis, the existence of an implant, and blood biochemistry data. The target antibiotic drugs were vancomycin (VCM), arbekacin (ABK), teicoplanin (TEIC), linezolid (LZD), and rifampicin (RFP), which are commonly used for the treatment of MRS in Japan. The breakpoint of ABK is not indicated in the CLSI. Therefore, the gentamicin (GM) breakpoint is used as a reference to determine the ABK breakpoint.

Culture method

Standard microbiological culture was performed using blood agar media (Eiken Chemical, Tokyo, Japan). The composition of the semifluid Gifu Anaerobic Medium (GAM) is 1 L containing peptone 10 g, soy peptone 3 g, proteose peptone 10 g, digestive serum powder 13.5 g, yeast extract 5 g, meat extract 2.2 g, liver extract 1.2 g, dextrose 3 g, potassium dihydrogen phosphate 2.5 g, sodium chloride 6 g, soluble starch 5 g, L-cysteine hydrochloride 0.3 g, sodium thioglycolate 0.3 g, vitamin K₁ 0.01 g, hemin 0.01 g, and agar 1.5 g, and it is adjusted to pH 7.2 ± 0.1. The composition of the blood agar medium is 1 L containing 23 g of peptone, 5 g of sodium chloride, 50 mL of sheep defibrinated blood, and 15 g of agar. Cultivation was performed at 35°C for 18 hours, and bacteria were allowed to grow for up to five days. Then, fresh isolates were cultured for 24 hours prior to drug susceptibility testing. The bacteria solution is adjusted using the prompt method. Independent single colonies of suspected staphylococci were harvested and suspended to form a bacterial solution. The MIC was determined using MicroScan WalkAway 96 Plus (Beckman Coulter, California) using the broth microdilution method and PC3.1J panel.

Statistical analysis

The MIC of each isolate was evaluated based on a 2 × 2 table comparison between orthopedic and respiratory samples using Fisher’s exact test and residual analysis. Differences in drug resistance between implant-related infections and other orthopedic infections were evaluated. A P value of <0.05 was considered statistically significant.

## Results

A total of 259 isolates were included in the study (89 orthopedic isolates and 170 respiratory medicine isolates). The original diagnosis of patients in the orthopedic group included 20 surgical site infections, 13 PJI (hip, 7; knee, 3; elbow, 2; toe, 1), eight osteomyelitis, six pyogenic arthritis, four purulent spondylitis, four osteosarcomas, four infectious atheromatosis, and 30 other diseases. Forty-seven isolates were from implant-related infections, including 13 PJI, seven spinal implantations, and 27 other implant-related surgeries. The remaining 42 isolates were obtained from infections unrelated to implants. The characteristics of the orthopedic and respiratory medicine groups are summarized in Table 1. No significant differences were seen between the two groups.

**Table 1:** Patient characteristics NS, not statistically significant; BMI, body mass index; CRP, C-reactive protein; ESR, erythrocyte sedimentation rate

	Orthopedic (N = 89)	Respiratory medicine (N = 170)	P value
Age, years	57 ± 10.1	67 ± 8.1	NS
Sex, male/female	52/37	88/82	NS
Height, cm	159.6 ± 5.2	156.2 ± 11.1	NS
Weight, kg	56.8 ± 6.4	53.4 ± 7.1	NS
BMI, kg/m^2^	22.2 ± 1.9	21.9 ± 8.2	NS
CRP, mg/dL	3.9 ± 3.3	3.5 ± 3.5	NS
ESR-60, mm/hour	36.7 ± 15	29.7 ± 8.9	NS

**Figure 1:**
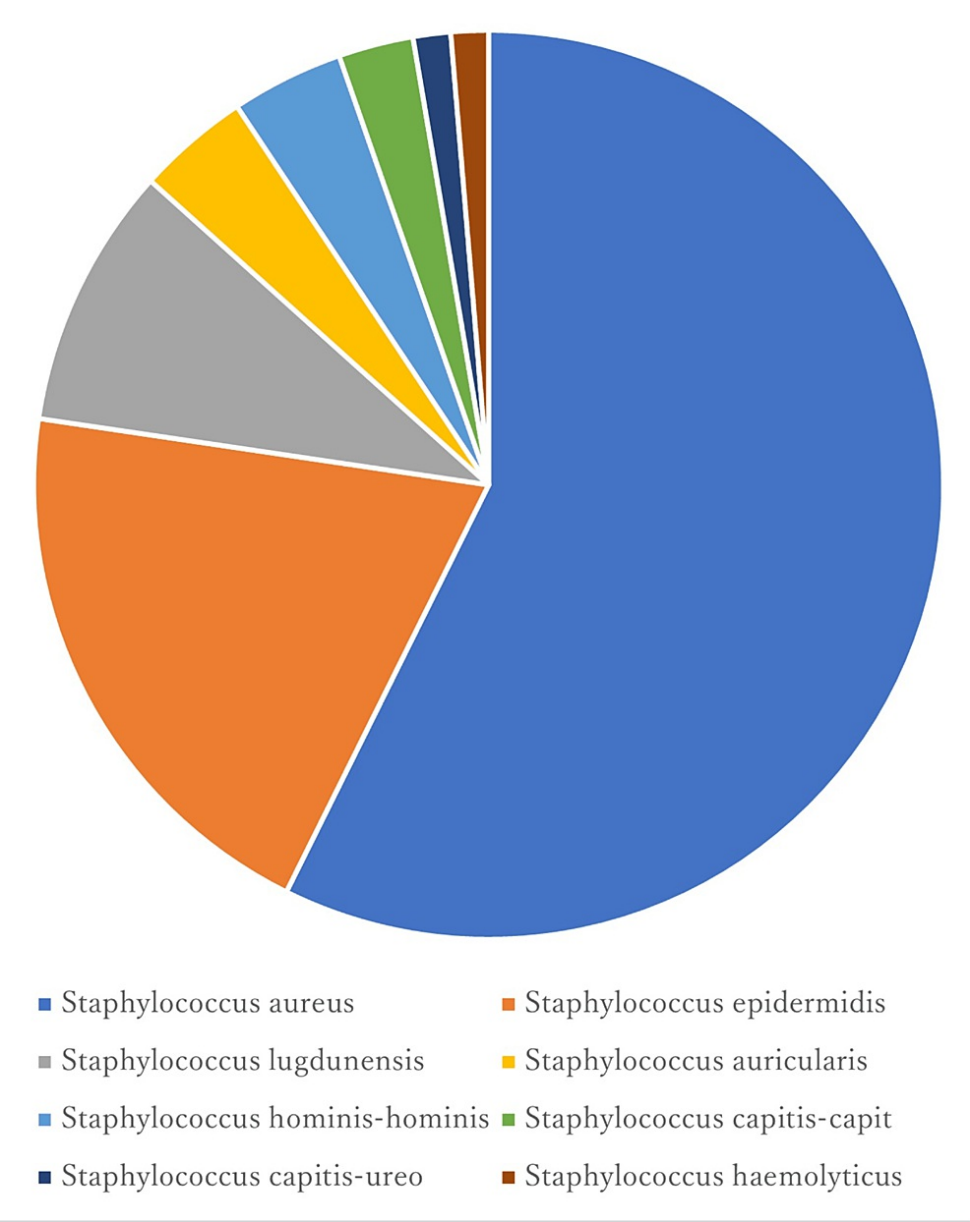
Percentages of Staphylococcus species among orthopedic isolates (N = 89) *Staphylococcus aureus *dominated all other *Staphylococcus* isolates with a percentage of 58%, followed by *Staphylococcus epidermidis *(20%) and *Staphylococcus lugdunensis* (9%).

The proportion of each *Staphylococcus* species identified from the orthopedic samples is shown in Figure 1.

*Staphylococcus aureus* was the most common species (58％), followed by *Staphylococcus epidermidis* (20%) and *Staphylococcus lugdunensis* (9%). Table 2 shows all the antibiotic MIC values of antibiotics of orthopedic and respiratory medicine isolates.

**Table 2:** MICs of antibiotic drugs in orthopedic and respiratory medicine isolates *P = 0.0078, **P < 0.001, ***P = 0.0058 MIC, minimum inhibitory concentration; MRCNS, methicillin-resistant coagulase-negative staphylococci; MSCNS, methicillin-sensitive coagulase-negative staphylococci; MRSA, methicillin-resistant *Staphylococcus aureus*; MSSA, methicillin-sensitive *Staphylococcus aureus*; VCM, vancomycin; TEIC, teicoplanin; ABK, arbekacin; LZD, linezolid; RFP, rifampicin

		Orthopedic				Respiratory medicine	
Antibiotic drugs	MIC (μg/mL)	MRCNS (%)	MSCNS (%)	MRSA (%)	MSSA (%)	MRSA (%)	MSSA (%)
VCM	<0.5	3 (13)	4 (31)	2 (13)	0* (0)	1 (3)	30* (22)
	1	8 (33)	7 (54)	10 (64)	32 (86)	29 (94)	100 (72)
	2	13** (54)	2 (15)	3 (20)	5* (14)	1** (3)	9* (6)
TEIC	<2	16 (68)	10 (77)	14 (97)	37 (100)	30 (97)	139 (100)
	4	4 (16)	2 (15)	1 (3)	0 (0)	1 (3)	0 (0)
	8	4 (16)	1 (8)	0 (0)	0 (0)	0 (0)	0 (0)
ABK	<1	20 (83)	13 (100)	8 (54)	27 (73)	22 (71)	111 (80)
	2	4 (17)	0 (0)	3 (20)	8 (22)	6 (19)	20 (14)
	4	0 (0)	0 (0)	2 (13)	2 (5)	3 (10)	8 (6)
	8	0 (0)	0 (0)	2 (13)	0 (0)	0 (0)	0 (0)
LZD	<2	24 (100)	13 (100)	14 (94)	25 (68)	30 (97)	123 (88)
	4	0 (0)	0 (0)	1 (6)	12 (32)	1 (3)	16 (12)
RFP	<1	19 (79)	13 (100)	15 (100)	37 (100)	28 (90)	138 (99)
	2	0 (0)	0 (0)	0 (0)	0 (0)	0 (0)	0 (0)
	>2	5*** (21)	0*** (0)	0*** (0)	0*** (0)	3 (10)	1 (1)

The number of isolates with a VCM MIC of <0.5 mg/L against methicillin-sensitive *Staphylococcus aureus* (MSSA) was significantly higher in respiratory medicine samples than those seen in orthopedic samples, while a MIC of 2 mg/L was significantly lower (P = 0.0078). A VCM MIC of 2 mg/L in methicillin-resistant coagulase-negative staphylococci (MRCNS) isolates was seen significantly more frequently in orthopedic samples than in respiratory isolates of methicillin-resistant *Staphylococcus aureus* (MRSA) (P < 0.001). There was no significant difference in the observation of higher MICs in ABK, TEIC, LZD, and RFP between orthopedic and respiratory isolates. When comparing MRCNS and other orthopedic staphylococci, the rate of MIC > 2 mg/L of RFP in MRCNS isolates was significantly higher (P = 0.0058). Table 3 shows the observed differences in drug resistance between implant-related samples and other orthopedic samples, showing a significantly higher rate of MRS in the implant-related samples (P = 0.021).

**Table 3:** Methicillin resistance in implant-related and implant-unrelated samples *P = 0.021 MRS, methicillin-resistant staphylococci; MSS, methicillin-sensitive staphylococci

	MRS	MSS
Implant-related sample	25^*^	21
Sample not related to implant	13	30^*^

Table 4 shows the distribution of VCM MIC in all MRCNS isolates; 77% of isolates with VCM MIC of 2 mg/L in MRCNS were detected in implant-related samples.

**Table 4:** MIC of VCM in orthopedic MRCNS isolates according to the presence or absence of an implant P = 0.6178 MIC, minimum inhibitory concentration; VCM, vancomycin; MRCNS, methicillin-resistant coagulase-negative staphylococci

	≦1 μg/mL	2 μg/mL
Implant-related sample	11 (85%)	10 (77%)
Sample not related to implant	2 (15%)	3 (23%)

## Discussion

We demonstrated that a VCM MIC of 2 mg/L of MRCNS from orthopedic isolates was significantly more frequent than that of MRSA from respiratory medicine isolates. In addition, orthopedic MRCNS isolates had a significantly higher rate of RFP drug resistance than other orthopedic isolates. Thus, MRCNS in orthopedic samples had a particularly higher rate of drug resistance.

Sensitivity to antibiotic therapy, including VCM for MRSA, has declined over time [5]. The MIC is reportedly increasing, and greater difficulty in the treatment of MRSA has been reported [4]. Several studies report that the treatment of staphylococci infections may fail if the MIC is >2 mg/L for VCM [6-8]. In addition, a meta-analysis reported that a VCM MIC of 2 mg/L was significantly associated with mortality in patients with MRSA infection [9]. In the present study, 77% of isolates with a VCM MIC of 2 mg/L against MRCNS were detected in implant-related samples. Therefore, patients with implant-related infections, such as PJI, are at risk of treatment failure with VCM alone. Single-agent antimicrobial therapy is reported to be particularly important as a risk factor for treatment failure in orthopedic device-related infections [10].

When considering the difficulty in treating implant-related infections, the relationship between MIC and biofilm formation is an important consideration. In biofilm isolates from orthopedic infections, many antibiotics (including VCM and RFP) have been shown to have an insufficient minimum biofilm eradication concentration [11]. Doroshenko et al. investigated the influence of sub-MIC VCM exposure in the biofilm of *S. epidermidis*, concluding that sub-MIC VCM increases biofilm tolerance through an extracellular DNA-based mechanism [12]. Similarly, Pasquaroli et al. reported that insufficient antibiotic pressure can induce a viable but non-culturable state in the *Staphylococcus *biofilm [13]. Therefore, antibiotic susceptibility, including a precise MIC value, should be determined in biofilm formative isolates from orthopedic infections.

Coagulase-negative staphylococci (CNS), particularly MRCNS, were isolated more frequently in implant-related samples than in other orthopedic samples. A previous study also demonstrated that implant-associated orthopedic infections involved more frequently commensal bacteria, such as CNS, than implant-free infections [14]. In addition, the incidence of a VCM MIC of 2 mg/L of MRCNS isolates was significantly higher than that of MRSA isolates in the present study. Regarding implant-associated infections in settings other than orthopedic, infective endocarditis is an important disease that directly correlates with mortality. García et al. reported that mortality was higher among patients who had isolates with a VCM MIC of ≥2 mg/L [15]. Therefore, close attention should be paid to the MIC of MRCNS in each isolate, particularly in implant-related infections. The CNS are believed to acquire resistance to multiple antibiotics through the formation of a biofilm around the implant. Although the number of isolates was not high, we detected seven cases of *Staphylococcus lugdunensis*. *Staphylococcus lugdunensis* has a pathogenicity equivalent to that of *S. aureus*, is difficult to treat, and is, therefore, associated with poor prognosis [16].

Regarding antibiotics other than VCM, there was no significant difference in MIC distribution in ABK, TEIC, LZD, or RFP between orthopedic and respiratory medicine isolates in the present study. RFP has been recognized as a useful drug for biofilm infection in orthopedic patients [17]. Although RFP-resistant staphylococci must be anticipated, particularly in patients with ≥3 previous surgical revisions [18], combined therapy is an effective regimen for PJI [19,20]. Nevertheless, the easy application of RFP should be restricted as it is an antitubercular drug. Particularly, we should note the risk of drug resistance acquisition due to widespread use. Our results showed that RFP exhibits good susceptibility both in orthopedic and respiratory medicine isolates. The exception, however, was seen to be MRCNS, with 21% of MRCNS from orthopedic isolates exhibiting resistance (>2 mg/L) to RFP, a significantly higher rate than that of MSCNS, MRSA, and MSSA from orthopedic samples. Therefore, it is also important to refer to the MIC of RFP for combination therapy with other drugs, particularly in cases involving MRCNS. A recent in vivo mouse model demonstrated the effectiveness of combination therapy using an LZD/RFP regimen for MRSA infection [21]. Indeed, in the present study, most of the orthopedic isolates, including CNS, showed good LZD susceptibility (MIC < 2 mg/L). By contrast, the MIC for TEIC was 8 mg/L (intermediate) in 16% of CNS. Although we could not investigate the MIC of CNS from respiratory medicine isolates, a recent study reported that 20% of all CNS isolates tested were non-susceptible to TEIC [22]. Therefore, the MIC in CNS might be important, not only for VCM but also for other antibiotics such as RFP or TEIC.

There are several limitations in this study. This study is a retrospective investigation, and the clinical outcome according to MIC was not evaluated as the outcome will be influenced by multiple factors. We used a single control group (respiratory medicine isolates), and it is possible that different results would be obtained from other groups, such as blood culture samples. In respiratory medicine isolates, we did not identify each CNS species as these isolates were usually identified as normal bacterial flora. Therefore, it was not possible to compare the MIC distributions in CNS between orthopedic and respiratory medicine isolates.

## Conclusions

In conclusion, the MIC of VCM in *Staphylococcus* species from orthopedic infections was higher than that of respiratory medicine isolates, particularly in MRCNS from implant-related samples. By contrast, there was no significant difference in the MIC of ABK, TEIC, LZD, and RFP between the two groups. MRCNS revealed a significantly higher rate of resistance for RFP than other orthopedic isolates. Thus, particularly in cases of MRCNS implant-related infection, it is important to refer to the MIC to ensure adequate antibiotic treatment.

## References

[1] Anagnostakos K, Schmitt C (2014). Can periprosthetic hip joint infections be successfully managed by debridement and prosthesis retention?. World J Orthop.

[2] Mortazavi SM, Vegari D, Ho A, Zmistowski B, Parvizi J (2011). Two-stage exchange arthroplasty for infected total knee arthroplasty: predictors of failure. Clin Orthop Relat Res.

[3] Hays MR, Kildow BJ, Hartman CW, Lyden ER, Springer BD, Fehring TK, Garvin KL (2023). Increased incidence of methicillin-resistant Staphylococcus aureus in knee and hip prosthetic joint infection. J Arthroplasty.

[4] Kullar R, Davis SL, Levine DP, Rybak MJ (2011). Impact of vancomycin exposure on outcomes in patients with methicillin-resistant Staphylococcus aureus bacteremia: support for consensus guidelines suggested targets. Clin Infect Dis.

[5] Steinkraus G, White R, Friedrich L (2007). Vancomycin MIC creep in non-vancomycin-intermediate Staphylococcus aureus (VISA), vancomycin-susceptible clinical methicillin-resistant S. aureus (MRSA) blood isolates from 2001-05. J Antimicrob Chemother.

[6] Dombrowski JC, Winston LG (2008). Clinical failures of appropriately-treated methicillin-resistant Staphylococcus aureus infections. J Infect.

[7] Hidayat LK, Hsu DI, Quist R, Shriner KA, Wong-Beringer A (2006). High-dose vancomycin therapy for methicillin-resistant Staphylococcus aureus infections: efficacy and toxicity. Arch Intern Med.

[8] Sakoulas G, Moise-Broder PA, Schentag J, Forrest A, Moellering RC Jr, Eliopoulos GM (2004). Relationship of MIC and bactericidal activity to efficacy of vancomycin for treatment of methicillin-resistant Staphylococcus aureus bacteremia. J Clin Microbiol.

[9] van Hal SJ, Lodise TP, Paterson DL (2012). The clinical significance of vancomycin minimum inhibitory concentration in Staphylococcus aureus infections: a systematic review and meta-analysis. Clin Infect Dis.

[10] Ferry T, Uçkay I, Vaudaux P (2010). Risk factors for treatment failure in orthopedic device-related methicillin-resistant Staphylococcus aureus infection. Eur J Clin Microbiol Infect Dis.

[11] Molina-Manso D, del Prado G, Ortiz-Pérez A, Manrubia-Cobo M, Gómez-Barrena E, Cordero-Ampuero J, Esteban J (2013). In vitro susceptibility to antibiotics of staphylococci in biofilms isolated from orthopaedic infections. Int J Antimicrob Agents.

[12] Doroshenko N, Tseng BS, Howlin RP (2014). Extracellular DNA impedes the transport of vancomycin in Staphylococcus epidermidis biofilms preexposed to subinhibitory concentrations of vancomycin. Antimicrob Agents Chemother.

[13] Pasquaroli S, Zandri G, Vignaroli C, Vuotto C, Donelli G, Biavasco F (2013). Antibiotic pressure can induce the viable but non-culturable state in Staphylococcus aureus growing in biofilms. J Antimicrob Chemother.

[14] Cuérel C, Abrassart S, Billières J, Andrey D, Suva D, Dubois-Ferrière V, Uçkay I (2017). Clinical and epidemiological differences between implant-associated and implant-free orthopaedic infections. Eur J Orthop Surg Traumatol.

[15] García de la Mària C, Cervera C, Pericàs JM (2015). Epidemiology and prognosis of coagulase-negative staphylococcal endocarditis: impact of vancomycin minimum inhibitory concentration. PLoS One.

[16] Shah NB, Osmon DR, Fadel H (2010). Laboratory and clinical characteristics of Staphylococcus lugdunensis prosthetic joint infections. J Clin Microbiol.

[17] Zimmerli W, Moser C (2012). Pathogenesis and treatment concepts of orthopaedic biofilm infections. FEMS Immunol Med Microbiol.

[18] Achermann Y, Eigenmann K, Ledergerber B (2013). Factors associated with rifampin resistance in staphylococcal periprosthetic joint infections (PJI): a matched case-control study. Infection.

[19] Kruse CC, Ekhtiari S, Oral I (2022). The use of rifampin in total joint arthroplasty: a systematic review and meta-analysis of comparative studies. J Arthroplasty.

[20] Leijtens B, Elbers JB, Sturm PD, Kullberg BJ, Schreurs BW (2017). Clindamycin-rifampin combination therapy for staphylococcal periprosthetic joint infections: a retrospective observational study. BMC Infect Dis.

[21] Yamada K, Namikawa H, Fujimoto H (2017). Clinical characteristics of methicillin-resistant coagulase-negative staphylococcal bacteremia in a tertiary hospital. Intern Med.

[22] Thompson JM, Saini V, Ashbaugh AG (2017). Oral-only linezolid-rifampin is highly effective compared with other antibiotics for periprosthetic joint infection: study of a mouse model. J Bone Joint Surg Am.

